# Molecular Modeling Study of Novel *Lancifolamide* Bioactive Molecule as an Inhibitor of Acetylcholinesterase (AChE), Herpes Simplex Virus (HSV-1), and Anti-proliferative Proteins

**DOI:** 10.3390/molecules27175480

**Published:** 2022-08-26

**Authors:** Malik Saadullah, Arshad Farid, Asad Ali, Muhammad Rashad, Faiza Naseem, Sheikh Abdur Rashid, Shakira Ghazanfar, Muhammad Yasin, Nosheen Akhtar, Mohammed S. Almuhayawi, Mohammed H. Alruhaili, Samy Selim

**Affiliations:** 1 Department of Pharmaceutical Chemistry, Government College University Faisalabad, Faisalabad 38000, Pakistan; 2 Gomal Center of Biochemistry and Biotechnology, Gomal University, Dera Ismail Khan 29050, Pakistan; 3 Department of Pharmaceutics, Faculty of Pharmacy, Gomal University, Dera Ismail Khan 29050, Pakistan; 4 National Institute for Genomics Advanced Biotechnology, National Agricultural Research Centre, Park Road, Islamabad 45500, Pakistan; 5 Department of Biological Sciences, National University of Medical Sciences, Rawalpindi 46000, Pakistan; 6 Department of Medical Microbiology and Parasitology, Faculty of Medicine, King Abdulaziz University, Jeddah 21589, Saudi Arabia; 7 Department of Clinical Laboratory Sciences, College of Applied Medical Sciences, Jouf University, Sakaka 72388, Saudi Arabia

**Keywords:** *Conocarpus lancifolius*, lancifolamide, *combretaceae*, anti-Alzheimer’s, anti-viral, cytotoxic, anti-proliferative, antibiotic-resistant bacteria, infection

## Abstract

*Combretaceae*, an immense family involving species (500) or genera (20), originates in tropical and subtropical regions. This family has evinced medicinal values such as anti-leishmanial, cytotoxic, antibacterial, antidiabetic, antiprotozoal, and antifungal properties. *Conocarpus lancifolius (C. lancifolius)* methanol extract (CLM) was prepared, then compound isolation performed by open column chromatography, and compound structure was determined by spectroscopic techniques (^13^C NMR, IR spectroscopy, ^1^H-NMR, mass spectrometry UV-visible, and 2D correlation techniques). Molecular docking studies of ligand were performed on transcriptional regulators 4EY7 and 2GV9 to observe possible interactions. Phytochemical screening revealed the presence of secondary metabolites including steroids, cardiac glycosides, saponins, anthraquinones, and flavonoids. The isolated compound was distinguished as lancifolamide (LFD). It showed cytotoxic activity against human breast cancer, murine lymphocytic leukemia, and normal cells, human embryonic kidney cells, and rat glioma cells with IC_50_ values of 0.72 µg/mL, 2.01 µg/mL, 1.55 µg/mL, and 2.40 µg/mL, respectively. Although no cytotoxic activity was noticed against human colon cancer and human lung cancer, LFD showed 24.04% inhibition against BChE and 60.30% inhibition against AChE and is therefore beneficial for Alzheimer’s disease (AD). AChE and LFD interact mechanistically in a way that is optimum for neurodegenerative disorders, according to molecular docking studies. Methanol and dichloromethane extract of *C. lancifolius* and LFD shows antibacterial and antifungal activity against antibiotic resistance *Bacillus subtilis, Streptococcus mutans, Brevibacillus laterosporus, Salmonella Typhi, Candida albicans,* and *Cryptococcus neoformans*, respectively. LFD shows antiviral activity against HSV-1 with 26% inhibition IP. The outcomes of this study support the use of LFD for cognitive disorders and highlight its underlying mechanism, targeting AChE, DNA-POL, NF-KB, and TNF-α, etc., for the first time.

## 1. Introduction

Neurodegenerative disorders are progressive and age dependent. Low protein levels of acetylcholine cause reported Alzheimer’s disease (AD) symptoms. Acetylcholine is broken down by acetylcholinesterase enzyme and is used in the brain [1]. Therefore, for the treatment of AD, one of the approaches is to use acetylcholinesterase inhibitors that reduce the acetylcholinesterase enzyme level so that acetylcholine remains in the brain [2]. Currently for AD treatment, four medicines are accessible to the market, and three are cholinesterase inhibitors, including rivastigmine, memantine, galantamine, and donepezil, which act as antagonists of N-methyl-D-aspartate (NMDA) receptor [3]. Rivastigmine and galantamine are naturally derived compounds among cholinesterase inhibitors, while memantine and donepezil are synthetic compounds. There are various disturbing side effects of all of the approved treatments [4]. Our group recently investigated *Combretaceae* family plants and *C. lancifolius* for better cholinesterase inhibitor activity against AChE enzyme, a distinct active compound [5].

Some similar studies were conducted that demonstrate strong cholinesterase inhibitory activity shown by some oleanane triterpenoids and ursane [6]. In fact, inflammation is associated with neurodegeneration. Another factor contributing towards neurodegeneration is oxidative stress. *C. lancifolius* and *Lancifolamide* belonging to the *Conocarpus* species show anti-inflammatory activity and sedative effects on the central nervous system (CNS). Insect repellent and feline attractant effect was shown by *Conocarpus* species, especially *C. lancifolius*, or there is association between anticholinesterase or insect repellent activities in some neurological disorders and AD treatment. All these effects represented the class of multi-targeted drugs by their cholinesterase inhibitors [5].

Herpes simplex virus (HSV) is an enveloped pathogen belonging to the Herpesviridae family that causes a wide spectrum of infections in humans. HSV type 1 (HSV-1) causes a range of infections in the mouth, mucous membranes, eyes, and pharynx, and is more common in immunocompromised patients [7]. This study is focused on the anti-Alzheimer’s and antiviral activity of *C. lancifolius* and lancifolamide (LFD). Several medicinal plants which contain flavonoids, anthraquinones, essential oils, and phenolic compounds possess good anti-viral (against HSV-1) and anti-Alzheimer’s activity [8].

*Conocarpus* genus consists of only two species namely, *C. erectus* and *C. lancifolius*. *C. lancifolius* is native to coastal and riverine areas of East Africa. *C. lancifolius* is an ornamental and fast-growing tree in sandy soils and semi-arid conditions. For polysaccharide, polyphenols and epicuticular waxes are secreted by two secretory ducts present on mature leaf [9]. *C. lancifolius* contains free anthraquinones, bound anthraquinones, saponins, flavonoids, and tannins [10].

This study is systematically designed by the combination of experimental and computational (docking) analysis to evaluate the cytotoxicity, anti-Alzheimer’s, and anti-viral activities of methanol and dichloromethane extract and novel compound from *C. lancifolius* (*Combretaceae*). The variety of normal and cancer cell lines were tried against the dichloromethane extract of *C. lancifolius* (CLD) and methanolic extract of *C. lancifolius* (CLM) extract and LFD to determine cytotoxic potential. Similarly, antiviral, anti-Alzheimer’s, and antibacterial potential was also determined by conducting anticholinesterase inhibitory activity, 2,2-diphenyl-1-picrylhydrazyl (DPPH), 2,2′-azino-bis(3-ethylbenzothiazoline-6-sulfonic acid) (ABTS), cupric reducing antioxidant capacity (CUPRAC) scavenging, and lipid peroxidation inhibitory activity, respectively. The different spectroscopic approaches were also applied to elucidate the structure of LFD.

## 2. Materials and Methods

### 2.1. Plant Material

The *C. lancifolius* was collected from the peripheries of Lahore, Punjab, Pakistan in August 2012 and identified by Professor Dr. Altaf Ahmad Dasti, Institute of Pure and Applied Biology, Bahauddin Zakariya University, Multan, Pakistan, and a voucher specimen (NO. WCL-291) was deposited for future reference in the herbarium.

### 2.2. General Experimental Procedures

Bruker Avance III-500 (Billerica, MA, USA), Shimadzu HPLC (CBM-20A, ^13^C-NMR: 125 MHz, Kyoto, Japan), MHz NMR (^1^H-NMR: 500 MHz, Heidolph and Buchi rotary evaporators (Schwabach, Germany), optical activity AA-55 Series, automatic polarimeters, molecular devices ELISA (San Jose, CA, USA), mass spectrometer with Zivak Tandem gold LC-MS/MS (Boca Raton, FL, USA), i.e., mass analyzer triple quadrupole, and Varian VNMRS-600 MHz NMR (1H-NMR:600 MHz or ^13^C-NMR:150 MHz, Knoxville, TN, USA), and for correlation HMBC and HMQC analyses were used to identify chemical features [11].

### 2.3. Extraction and Isolation

Aerial parts of plant were dried at room temperature and 900 g powdered plant material was obtained. Then, powdered *C. lancifolius* was extracted with 4 L dichloromethane along with methanol, i.e., 7 days × 3 times successively. Solvents were evaporated after filtration to obtain dry extract under a vacuum using a rotary evaporator; this process obtained 22.0 g methanol extract and 27.5 g dichloromethane extract. By utilizing 5 × 150 cm silica gel column crude dichloromethane extract was then fractionated [12]. Spot checking on thin-layer chromatography and close fractions were combined, and 162 fractions were obtained. Further purifications by thin layer chromatography (TLC), preparative column chromatography, and high performance liquid chromatography (HPLC) were carried out on fractions for isolation of pure compound fraction [13].

#### Lancifolamide (LFD)

A yellow-brown amorphous solid [α]_D_^20^: +13.3 with CHCl_3,_ *c* 0.3 g/100 mL was obtained and is referred to as LFD. Using HPLC, a fraction was chromatographed (5 μm PREP-ODS Shim-pack (H) kit (Shimadzu, Kyoto, Japan), 250 × 4.6 mm, C18 column), and the extract was correlated with dichloromethane/acetone (7:3) gradient eluted with 100% methanol to provide a pure compound, i.e., 10 mg [14].

### 2.4. Analysis of High-Performance Liquid Chromatography or HPLC

Sample filtration was performed by membrane of Millex-HV PVDF 0.45 μm (Millipore, New Bedford, MA, USA). On a Shimadzu chromatographer (Kyoto, Japan), HPLC analysis was carried out with a ternary pump or LC-20AT Shimadzu (Kyoto, Japan) along with a DAD, i.e., Diode Assay Detector, or SPD-M20A Shimadzu (Kyoto, Japan), then performed on an analytical column (Phenomenex^®^ 5 μm ODS 100 A 250 mm × 4.60 mm) antecede by a guard C18 column (5 μm, 2.0 cm × 4.0 mm) belonging to Phenomenex (Torrance, CA, USA). Version 1.25 of the LC solutions software was operated for data processing with gradient chromatographic conditions, with a mobile phase comprising water and acetonitrile. Initially, 2:8 *v/v* of acetonitrile/water was utilized as a mobile phase that increased gradually up to 8:2 *v/v* of acetonitrile/water in 30 min at a 1ml/min flow rate. Before use, the freshly prepared mobile phase was degassed by a sonication process at 25 °C of constant column temperature along with 20 μL used injection volume. Monitored UV spectrum ranges from 450 to 200 nm [15]. Methanol to prepare extracts or standard solutions was used as a solvent. Standard solutions were available in distinct concentrations, i.e., 500.0, 250.0, 150.0, 100.0, 25.0 and 12.5 μg/mL, and compound concentration was 2000 μg mL^−1^ [16].

### 2.5. Molecular Docking

The ligand–target protein interaction studies were accomplished by molecular docking using a transcriptional regulator 4EY7 (Human acetylcholinesterase co-crystal structure (AChE) [17] complex with donepezil; Alzheimer’s Disease), 2GV9 (HSV-1 DNA polymerase (DNA-POL) [18,19], and LFD (ligand). The 3D-conformation of LFD along with corresponding standard inhibitors GLM (Galantamine) and ACV (Acyclovir) were produced by module of Sybyl-X1.3/SKETCH [20] that followed energy minimization as stated by force field (Tripos) along with the atomic charge of Gasteigere Hückel [20]. Likewise, 4EY7 (human acetylcholinesterase co-crystal structure (AChE) complex with donepezil) and 2GV9 ((HSV-1 DNA polymerase (DNA-POL)) were retrieved from the Protein Data Bank (PBD).

All ligands and targets were prepared for molecular docking studies using structural preparation tools implied in the *SYBYL-X1.3* biopolymer module. The addition of missing hydrogens, charges, or atom types was applied or assigned in accordance with a force field such as AMBER 7 FF99, and was followed by energy minimization according to the Powell algorithm with 0.5 kcal mol^−1^ Å^−1^ convergence gradient for 1000 cycles. Molecular docking studies were performed with the SybylX-1.3 and Surflex-Dock modules [21]. Experimentally determined donepezil with its active conformation in the 4YE7 active site was used to define the potential binding pocket for the idealized active site of protomol generation. Parameters determining the protomol extent retained at a default such as threshold = 0.50 or bloat = 0). ACV, LFD, and GLM compounds were docked individually in the corresponding molecular target binding site with the molecular alignment algorithm as “whole”, and 20 top docked poses were saved for each individual ligand.

### 2.6. Antioxidant Assays

#### 2.6.1. Scavenging Activity of DPPH Assay of Free Radical

By utilizing scavenging activity of DPPH assay, i.e., 2,2-diphenyl-1-picrylhydrazyl of the free radical method exemplified by Blois, we determined the scavenging activity of free radical extracts and isolated pure terpenoids. The absorption characteristic of DPPH at 517 nm means it is considered a free radical (stable). By this method, pure compounds are extracted and isolated and the decreased absorption and antioxidant activity of extracts are determined. When a DPPH radical combines with an antioxidant its absorption decreases. Standards (BHA (Butylated hydroxyanisole), BHT (Butylated hydroxytoluene), α-tocopherol), isolated pure compounds, 40 µL of each of the extracts, and 160 µL of 0.1 mM DPPH solution were added to a 96-well plate at different concentrations. Solution absorption was estimated at 517 nm after 30 min duration incubated in darkness at 517 nm [11]. The experiment was performed thrice (n = 3) to show the reproducibility of the results.

#### 2.6.2. Scavenging Activity of ABTS Cation Radical Assay

To determine the scavenging activity, the potential of the ABTS cation radical for samples or extract, depending on the modified protocol of this method, was used. By an ABTS stock solution reaction (7.4 mM) with 2.45 mM (1:1, *v/v*) potassium persulphate solution, keeping the mixture overnight for 12–16 h at room temperature in the dark, then an ABTS cation radical, i.e., 2,2′-azino-bis(3-ethylbenzothiazoline-6-sulphonic acid), was prepared. Cationic ABTS radical solution was diluted until it gave an absorbance value, with distilled water, ranging between 1.0 and 1.2. Then, 1 mL of diluted aliquot sample was added to 50 mL of each of the above radical solutions, and was preserved or prepared in 60 min with light by using a spectrophotometer absorbance that was measured at 734 nm, and standards BHT, BHA, and α-tocopherol were used [11]. The experiment was performed thrice (n = 3) to show the reproducibility of the results.

#### 2.6.3. Assay of Inhibitory Activity of Lipid Peroxidation

By using the model system of β-carotenelinoleic acid, an evaluation was performed to estimate the antioxidant activity and the isolated pure terpenoids. In an emulsifier mixture of 100 μL linoleic acid, Tween 40 (800 μL) or β-Carotene (1 mg) in chloroform (2 mL) was added. Standards or isolated pure compounds transferred into a 960well plate at different concentrations along with 160 μL of the emulsion mixture. By vigorous shaking, distilled water (200 mL) was added which was saturated with oxygen. Using the spectrophotometer, zero-time absorbance at 490 nm was measured and each 40 μL emulsion was added. Absorptions at 490 nm were measured when the emulsion system was incubated at 50 °C for 2 h. Preparation of blank devoid β-carotene for background subtraction was performed. Standards BHT, BHA, and α-tocopherol were used [11]. The experiment was performed thrice (n = 3) to show the reproducibility of the results.

#### 2.6.4. CUPRAC (Cupric Reducing Antioxidant Capacity) Assay

By this method, along with the reducing power of copper, the antioxidant activity of the samples was determined. From *C. lancifolius,* 67 μL of each sample (extract, standard or isolated pure compounds) was added to 96-well plates at different concentrations and 0.01M CuCl_2_·2H_2_O (61 μL), alcohol solution (61 μL) of neocuproine (7.5 × 10^−3^ M), aqueous buffer of ammonium acetate (61 μL) at pH = 7 was added to a 96-well plate, and then at 450 nm absorbances were measured after 30–60 min. Standards BHT, BHA, and α-tocopherol were used [11]. The experiment was performed thrice (n = 3) to show the reproducibility of the results.

### 2.7. In Vitro Cytotoxicity of Isolated Compound

By sulforhodamine B procedure (SRB), the in vitro determination of cytotoxic potential for each isolated compound or *C. lancifolius* extract was performed [22]. Tests of samples diffused in a stock solution of 4 mg/mL (DMSO) were performed in triplicate containing a DMSO final concentration (0.5%), and ellipticine was utilized as positive and DMSO (0.5%) as negative controls, correspondingly [23]. Cancer cell lines ASK, P-388, MCF-7, T24, and Hek 293 were grown in 96-well plate in media (RPMI-1640) or MEM (minimum essential medium along with L-glutamine or Earle’s salt) with FBS 10 % (Fetal Bovine Serum), and Lu-1 was grown in MEM along with 5% FBS. For 72 h the drug was disclosed at 37 °C (P-388 for 48 h) in air with CO_2_ (5%) and 100% relative humidity. Cells were fixed with 10% trichloroacetic acid at the final concentration and then stained with 0.4% SRB in 1% acetic acid [24]. We then bound, dried, or solubilized the stain with 10 Mm trizma base and then the unbound dye was removed by washing. Absorbance read at 510 nm utilizing a FLUO star optima BMG plate reader. The experiment was performed thrice (n = 3) to show the reproducibility of the results.

### 2.8. Anticholinesterase Activity Assay

By the Ellman method, sample inhibition of BChE or AChE enzymes was determined. Amounts of 20 μL of either AChE or BChE enzyme solutions, phosphate buffer solution (130 μL) at pH 8, and 10 μL of each sample at different concentrations were added to 96-well plates and absorbance of zero-time was measured at 412 nm. Amounts of 20 μL DTNB, and 20 μL of acetylthiocholine iodide, i.e., AcTChI, or butyrylthiocholine iodide i.e., BuTChI, solutions were added after a 15-min incubation period at ambient temperature, then at 412 nm a second measurement after 10 min was carried out. Then, at room temperature this process was executed. Ethanol was used as a blank and galanthamin was used as standard. Anticholinesterase activity tests were executed in triplicate [11]. The experiment was performed thrice (n = 3) to show the reproducibility of the results.

### 2.9. Virucidal Compound Effects or Extracts on HSV-1

Viral suspension was performed at 37 °C, and samples were inoculated for 1 h with fractions (6 mg/mL) [25]. The mixture was then allowed for 1 h to infect the Vero cells. It was removed after that, washed with PBS wells at pH 7.4, then inoculated with methylcellulose/medium 0.5% for 72 h [26]. Medium was aspirated after 3 days then infected cells fixed with formaldehyde (10%), stained with 1% crystal violet, and plaques were counted [27]. Wells overlaid without or with the sample (tested) or medium were utilized as controls. The % inhibition of plaque lysis formation was calculated as follows:

$$\mathrm{IP}=\frac{\left[\left(\mathrm{mean}\;\#\;\mathrm{of}\;\mathrm{plaques}\;\mathrm{in}\;\mathrm{control}\right)\;–\;\left(\mathrm{mean}\;\#\;\mathrm{of}\;\mathrm{plaques}\;\mathrm{in}\;\mathrm{sample}\right)\right]}{\mathrm{mean}\;\#\;\mathrm{of}\;\mathrm{plaques}\;\mathrm{in}\;\mathrm{control}}\times 100$$

The experiment was performed thrice (n = 3) to show the reproducibility of the results.

### 2.10. Antimicrobial Studies

Whatman filter paper, i.e., the paper-disc method, possessing discs of 6.0 mm diameter, 250 µg/mL of Ampicillin (standard drug), 1.0 mg/mL of sample, along with 3 µL of injected sample were added on all paper discs with 6.0 mm diameter, and were infused with an antimicrobial solution kept on the culture medium. Antimicrobials were also applied to each disc after placing it on medium. Plates containing a single medium layer with a thickness of 2 mm were utilized for these tests. Afterwards, a zone of inhibition was noted [28]. Bacteria and fungi were isolated from ear discharge and wound swabs.

### 2.11. Statistical Analysis

Statistical analysis was performed using a post hoc test in Graphpad-prism version #9 for inter-group comparison. The analyses of results were performed by one-way or two-way analysis of variance (ANOVA) by a Bonferroni post-test. The data were indicated as mean ± SD, and *p* values < 0.05 were considered as statistically significant.

## 3. Results

### 3.1. Extraction and Isolation

**Figure 1:**
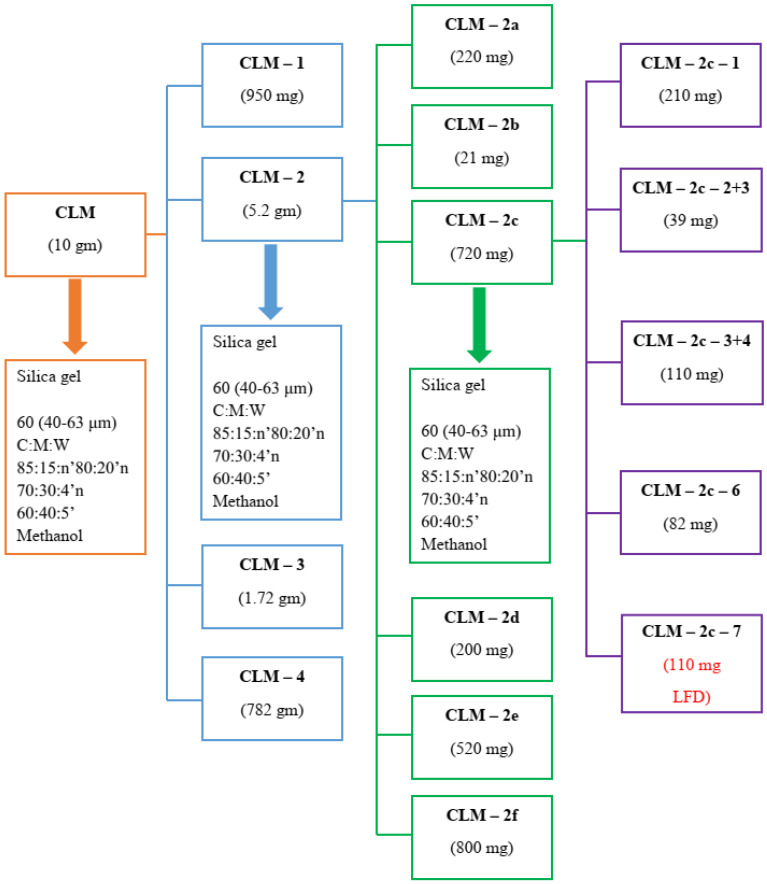
LFD isolation from *C. lancifolius* methanol extract.

The complete isolation scheme of LFD from 10 gm of methanol extract of *C. lancifolius* is given in Figure 1, the fraction CLM-2c-7 of 110 mg is the isolated pure compound LFD.

### 3.2. Analysis of High-Performance Liquid Chromatography or HPLC

**Figure 2:**
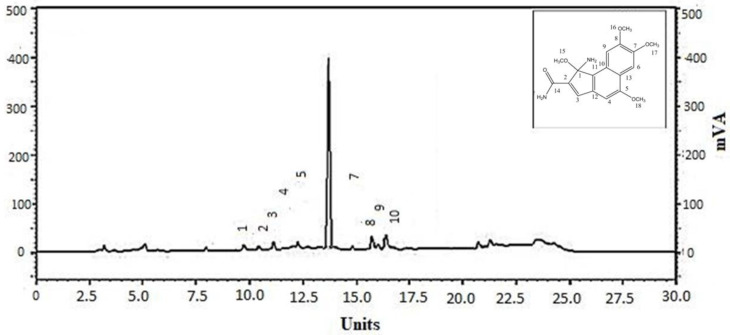
HPLC chromatogram and chemical structure of isolated compound of *C. lancifolius* “LFD”. HPLC data showed a single prominent peak of LFD.

The HPLC of the separated fraction of LFD from the methanol extract of *C. lancifolius* shows the prominent peak for LFD. The HPLC chromatogram is given in Figure 2.

### 3.3. Structural Elucidation

**Figure 3:**
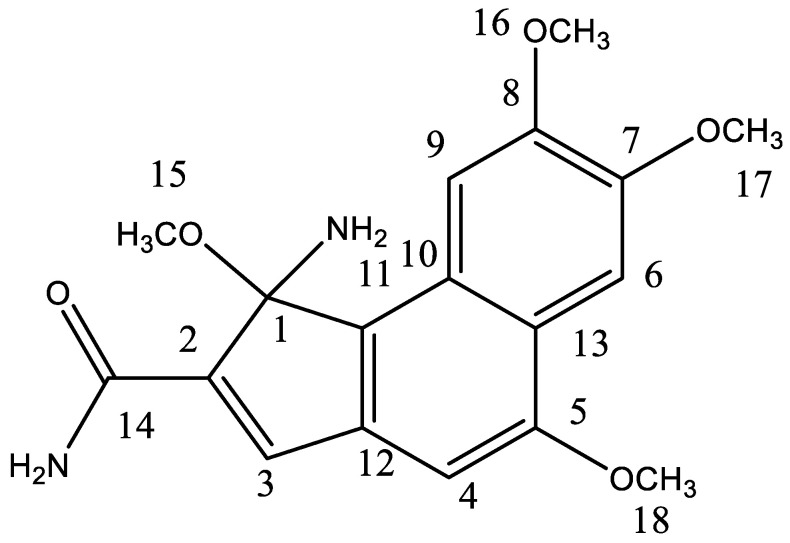
Chemical structure of lancifolamide.

The chemical structure of lancifolamide is shown in Figure 3.

**Physical Data:** Amorphous colorless solid, **Quantity:** 210 mg, **Melting point:** 210 to 220 °C, **Ultraviolet** *λ_max_*: 210, 230 nm, **IR** (KBr) ν_max_: 2867, 3657, 1624, 2931 cm^−1^, **Proton NMR:** utilizing 600 MHz, Proton resonate *δ* 5.695 (^1^H, d containing *J* = 2.3 Hz, H-6), 6.63 (^1^H, s, H-2), *δ* 5.880 (^1^H, d constituting *J* = 2.6 Hz for H-3), *δ* 4.771 (^1^H, s, H-4), *δ* 3.984 (^3^H, for -OCH_3_), **^13^C-NMR:** utilizing 150 MHz, Signals at *δ* 113.55 (C-8), *δ* 109.50 (C-1), *δ* 110.4, *δ* 139.35 (C-9), *δ* 109.24 (C-4), *δ* 109.91 (C-7), 108.20 (C-5), 138.54 (C-3), *δ* 109.40 (C-6), *δ* 138.20 (C-2), **EI-MS:** (rel. int.) *m/z* 162.5 (56), 344 (100), 177 (83), 148.5 (65), 69 (73),(87.5), 135 (33), 120.5 (45), 94.5 (64), **HR-EI-MS:** 344.14 (for C_18_H_20_ N_2_O_5_, 344.14)

LFD was obtained as an amorphous powder from the methanolic extract of *C. lancifolius*. An IR spectrum of LFD indicated a carbonyl group at 1723 cm^−1^ by. At 3654 cm^−1^, the absorption signals showed the presence of an N-H group in the molecule. Absorption of 1634 cm^−1^ occurred in the molecule owing to the amide group. Sp^3^ or Sp^2^ C-H stretching was present at 2931 or 2867 cm^−1^ [15].

The proton NMR spectrum of lancifolamide shows signals at δ6.636 ppm or signals in the region of downfield exhibits that a molecule functional group, such as N-H, is present. Signals at δ6.633 for ^1^H, d *J* = 2.6 Hz, or 6.857 for ^1^H, d, *J* = 3.1, 2.4 Hz exhibits alkene’s molecular nature.

**Figure 4:**
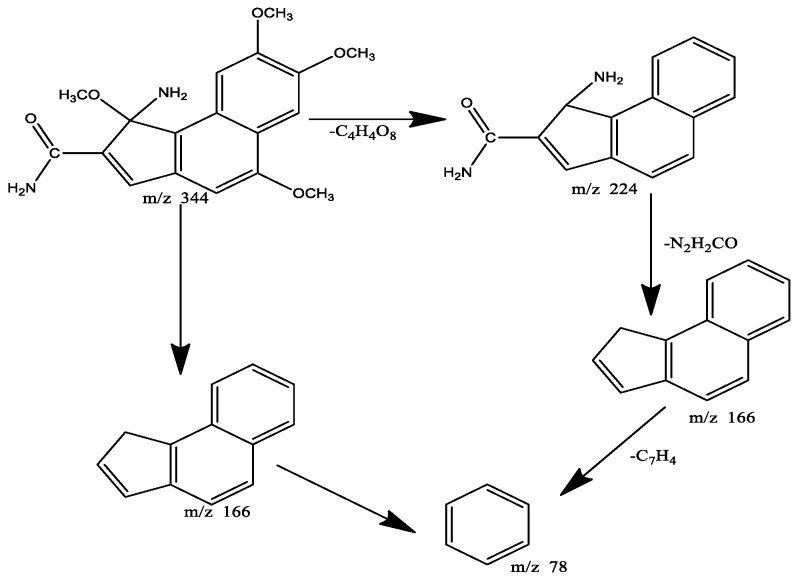
Mass fragmentation of LFD.

The molecular formula was C_18_H_20_N_2_O_5_ determined at m/z 344 by HR-EI-MS consisting of an ion peak conducive to C_18_H_20_N_2_O_5_:344.14. Depth or wide band (^13^C-NMR) lancifolamide compound spectra showed 18 carbon signals in the molecule: 4 methoxy, 4 methane, and 10 quaternary carbons. Signals at δ137.4, 132.4, 136.0, and 133.7 were downfield and indicate the existence of alkene carbon. The correlation of carbon and hydrogen of the novel compound was performed by HMBC and HSQC analysis. The mass fragmentation of LFD is shown in Figure 4.

The LFD compound 1-amino-1,5,7,8-tetramethoxy-1-H-cyclopenta naphthalene-2-carboxamide, a novel or natural product, was confirmed by the obtained values of physical or spectral data. The name lancifolamide was assigned on a species basis.

### 3.4. Molecular Docking

Experimental study results revealed that tested hit LFD showed the best fit in the active pocket of 4EY7 and 2GV9. Knowledge of the 3D structure of AChE and DNA-POL is clear as it exhibited key molecular interactions important for forming a ligand–protein complex. Surflex molecular docking may provide a useful understanding for key molecular interactions important for differentiating the binding affinities of ligands towards a targeted protein. Surflex scoring function CScore comprises distinct scores, i.e., Crash, Chem, G, PMF, D-score, and G score [29]. In CScore terms, entire energies or docking scores of pretentious ligand conformity, together with amino acid remnants, participated in the hydrogen bonding interactions tabulated in Table 1. Standard docking compound scores of GLM and the tested molecule LFD for AChE were 5.64 and 6.63, respectively, indicating that LFD contains a higher binding potential towards AChE than GLM. Similarly, ACV and LFD also display similar values of docking scores (CScore: 10.32 and 9.09, respectively) suggesting that the tested compounds exhibit almost similar binding affinity towards DNA-POL as that of the standard compound ACV. Among cell cycle regulators, the compound LFD displayed highest the docking scores against BCL-XL, BCL2, and caspases-3 of values 6.72, 4.18, and 4.78, respectively, which indicates that LFD may induce its cytotoxic activity via the inhibition of anti-apoptotic proteins. Among all studied proteins, compound LFD has demonstrated the lowest docking scores for NF-kB and TNF-α (Table 2).

**Figure 5:**
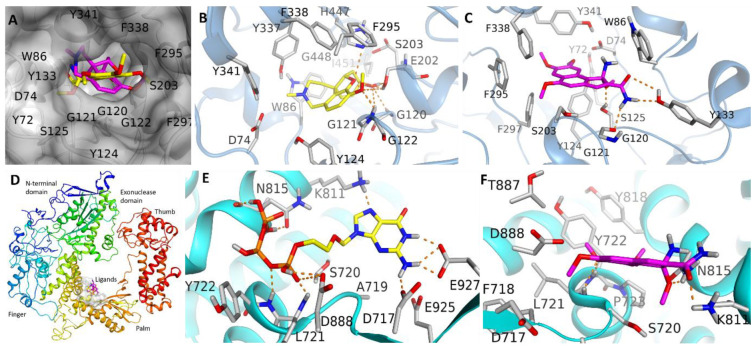
Comparison of binding mode of tested compound LFD with the standard compounds Galantamine (GLM) and Acyclovir (ACV) bind to their respective targets. (**A**) LFD (magenta) and GLM (yellow) superposed in the same binding cavity of AChE enzyme. Best docking generated poses of selected ligands in AChE (**B**) GLM-AChE and (**C**) LFD-AChE. Superposition of LFD (magenta) and ACV (yellow). (**D**) Overall atomic model of LFD (**E**,**F**) ACV and LFD bonded to DNA-POL, respectively. Predicted bonding interactions of hydrogen illustrated in dashed lines (yellow).

In an AChE-bonded system, GLM and LFD acquire similar modes of interaction within the active site of the protein and the residues Asp74, Trp86, Gly120, Gly121, Gly122, Tyr124, Glu202, Ser203, Phe295, Phe297, Tyr337, Phe338, Tyr341, His447, Gly448, and Ile451 are the key residues constituting the ligand binding site (Figure 5). The same binding pocket has previously been reported to serve as a ligand binding site in AChE [30]. The top poses of the standard compound (GLM-AChE) were found to be penetrated deep into the binding cavity to establish a network of several molecular interactions such as hydrogen bonding with the active site residues Gly120, Gly121, and Gly122. A similar pattern of H-bond interaction was observed in the case of LFD-bonded systems. Unlike GLM-AChE, the -NH_2_ group of LFD compound donated a couple of H-bonds to the nearby residue Gly120 and Tyr133 and accepted one H-bond from the side chain of Ser125. In addition, the methoxy groups of LFD extend towards a shallow Phenyl-rich cavity to establish hydrophobic interactions with Phe295, Phe297, and Phe338. In a GLM-AChE system, no hydrophobic interaction was observed that showed slightly higher docking scores of LFD towards AChE than GLM. Similarly, LFD demonstrated almost similar binding affinity towards the DNA-POL as the corresponding standard compound ACV. As depicted in Figure 5, both ligands occupy the same binding site situated at the palm region of DNA-POL. In the DNA-POL-ACV complex, the triphosphate of ACV establishes at least nine H-bonds with nearby residues including K811, N815, and D888. Conversely, LFD forms five H-bond interactions with common residues K811 and N815. Involvement of these residues in DNA binding and inhibition are well discussed previously [31,32]. In addition, the methoxy moiety of LFD extends towards the negatively charged oxygen atoms of D717 to develop *van der Walls (vdW)* interaction. Another methoxy group of LFD approaches Tyr818 in a perpendicular way to establish sigma–pi stacking. Despite additional hydrophobic and sigma–pi contacts in the LFD-DNA-POL system, higher binding affinity of ACV in DNA-POL arises due to its greater number of electrostatic interactions.

**Figure 6:**
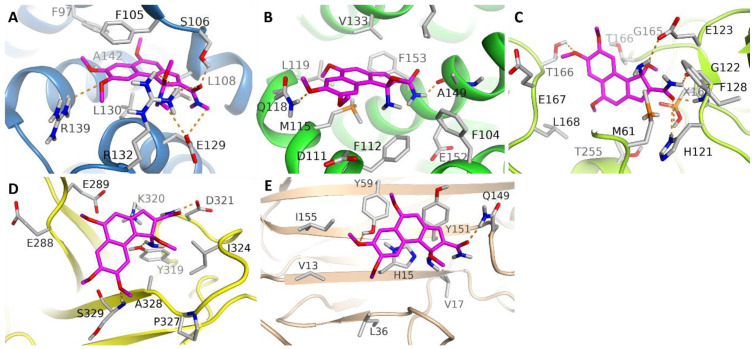
Top ranked docking conformations of our tested hit in selected proteins (**A**) LFD-BCL-XL, (**B**) LFD-BCL-2, (**C**) LFD-Caspases-3, (**D**) LFD-NF-kB, and (**E**) LFD-TNF-α. Predicted bonding interactions of hydrogen illustrated in dashed lines (yellow).

Among the cell cycle transcriptional regulator 2GV9 (HSV-1 DNA polymerase (DNA-POL), the compound LFD showed moderate to higher binding affinity towards BCL-XL, BCL2, and Caspases 3, respectively. Graphical analysis of top-ranked docking poses of LFD in BCL-XL reveals that the LFD establishes six H-bond interactions with residues Ser106, Glu129, Arg132, and Arg139. The benzyl moiety of Phe105 lies parallel to the benzene ring of LFD to develop pi–pi interactions. The methyl moiety from the backbone of Leu130 is oriented toward the benzene ring of LFD to establish sigma–pi stacking. Hence, as a consequence of the variety of molecular interactions, LFD displays higher binding affinity towards BCL-XL than that of other studied proteins. In the case of anBCL2-bonded system, LFD forms a couple of H-bonds with residues Gln118 and Ala149. Although a compound LFD produces six H-bonds in the LFD-Caspases 3 system, relatively lower docking scores are the consequence of the absence of sigma–pi and pi–pi interactions. Conversely, LFD demonstrated the weakest binding affinities towards NF-kB and TNF-α (Figure 6). The obtained docking results are in strong correlation with these experimental findings.

### 3.5. Antioxidant Assays

#### 3.5.1. Scavenging Activity of DPPH, ABTS, and Lipid Peroxidation Inhibitory Activity

The IC_50_ values of the *C. lancifolius* extracts (μg/mL) are given in Table 3; this reveals that the methanol extract of *C. lancifolius* shows good activity, as DPPH assay with 162.21 IC_50_ value, ABTS assay with 69.16 IC_50_ value, and LFD shows good lipid peroxidation inhibition, i.e., 140.91 IC_50_ value. This reveals that the *C. lancifolius* and LFD have antioxidant potential.

#### 3.5.2. CUPRAC (Cupric Reducing Antioxidant Capacity) Assay

In this assay, by the help of the reducing power of copper, the antioxidant potential of compound is determined. Results in Table 4 reveal that the antioxidant potential of the extract and novel compound is high at higher concentration. High antioxidant activity is favorable for the anti-Alzheimer’s activity.

### 3.6. Cytotoxic Assay

ED_50_ concentrations less than 20 µg/mLfor extracts or less than 4 µg/ mL for pure compounds are considered active. P388 of murine lymphocytic leukemia, Col-2, i.e., human colon cancer, MCF-7 of human breast cancer, and Lu-1 of human lung cancer showed significant results for methanol extract i.e., 2.06, 8.14, 0.85, and 7.64, and for lancifolamide, p-388 and MCF-7 showed 2.01, and 0.72 while no response (ED_50_ > 20 µg/mL) observed for COL-2 and Lu-1. The normal cells, i.e., ASK of rat glioma cell, Hek293 for noncancerous human embryonic kidney cell, showed 5.40 and <4 for methanol extract and 2.40 and 1.55 for lancifolamide [33,34]. The results shown in Table 5 reveal that LFD significantly increased the death of murine lymphocytic leukemia and human breast cancer cells when compared to ASK and Hek293 as normal cells. Hence it has significant cytotoxic activity.

### 3.7. Anticholinesterase Activity Assay

Anticholinesterase, along with the antioxidant activity of the extracts and isolated LFD (novel), were scrutinized. The AChE and BChE inhibition or antioxidant activity results of the methanol extract were found to be better by contrast than the dichloromethane extract due to the presence of phenolic constituents along with flavonoids [11]. The lancifolamide shows 60.30% AChE inhibition with 34.62 μg/mL IC_50_ value, shown in Table 6.

### 3.8. Virucidal Effects of Compound and Extracts on HSV-1

The methanol and dichlomethane extract of *C. lancifolius* and novel compound LFD shows 17, 13, and 26% inhibition with IC_50_ values less than 100. This reveals that the LFD possesses good antiviral activity. Results are shown in Table 7.

### 3.9. Antimicrobial Studies

The results in Table 8 reveal that the CLD, CLM, and LFD have high antibacterial and antifungal activity. The LFD shows high activity against *S. mutans, C. albicans,* and *C. neoformans,* respectively.

## 4. Discussion

*C. lancifolius* is one of the most important plants of the genus *Conocarpus,* belonging to the family *Combretaceae*. The novel compound seems to be biosynthesized by the shikimic acid pathway. According to different research studies, the genus conocarpus has showed significant therapeutic properties such as anticancer, antiprotozoal, antibacterial, and anti-leishmanial [9]. Experimental study showed that tested hit LFD have significant antiviral and cholinesterase inhibition potential when compared to standard compounds (GLM and ACV). Three-dimensional structural base knowledge about AChE and DNA-POL exhibited key molecular interactions. Surflex molecular docking provides the scoring function. CScore includes docking scores and energies which participate in hydrogen bonding interactions. The standard docking compound scores GLM and tested molecule LFD for AChE are 5.64 and 6.63, respectively, indicating that LFD has a higher binding potential towards AChE than GLM. ACV and LFD also displays similar docking score values (CScore: 10.32 and 9.09, respectively) suggesting that tested compounds exhibit the same binding affinity towards DNA-POL, such as the ACV standard compound. Compound LFD displayed the highest docking scores against BCL-XL, BCL2, and caspases-3 of values 6.72, 4.18, and 4.78, respectively, for cell cycle regulators representing LFD-induced cytotoxicity via anti-apoptotic protein inhibition. Among all the studied proteins, the compound LFD has demonstrated the lowest docking scores NF-kB and TNF-α. All these experimental studies show the anti-Alzheimer’s, antiviral, and cytotoxic effects of LFD.

Comparison is observed between the binding mode of the tested compound LFD with GLM and ACV standard compounds bound to their respective targets. LFD and GLM were superposed in the same binding cavity of AChE enzyme. The best docking-generated poses of selected ligands occur in AChE, GLM-AChE, and LFD-AChE. LFD and ACV show superposition and binding to DNA-POL, respectively.

Among cell cycle regulators, high binding affinity of the LFD compound towards BCL-XL (Phe105 benzyl and methyl moiety from Leu130 backbone develop pi–pi and sigma–pi interactions when they lie parallel or oriented towards an LFD benzene ring), BCL2 (LFD forms H-bonds with residues Gln118 and Ala149), and Caspases 3 (LFD compound establishes six H-bonds) is discussed. Graphical analysis shows six H-bond interactions of top-ranked LFD in a BCL-XL docking pose with different residues Ser106, Glu129, Arg132, or Arg139. Conversely, LFD exhibited the weakest binding affinities towards NF-kB and TNF-α.

Alzheimer’s disease is a neurodegenerative, age-dependent disease [35]. Oxidative stress is the proximal event in Alzheimer’s disease pathogenesis. The mitochondrial dysfunction, amyloid-β-mediated processes, transition metal accumulation, and genetic factors such as apolipoprotein E and presenilins are responsible for redox imbalance [36,37]. One of the methods for the treatment of Alzheimer’s is oxidative balance, i.e., reduction of free radical species formation. In the present study, four in vitro antioxidant assays (DPPH, ABTS, CUPRAC, and lipid peroxidation inhibition) were performed for CLD, CLM, and LFD. The LFD and CLD shows less antioxidant potential in DPPH, ABTS, and β-Caroten assays, but CLM shows good antioxidant potential in the ABTS assay with 69.16 IC_50_ value. These results reveal that the methanol extract contains phenolic and flavonoidal compounds in large quantities. The presence of flavonoidal and phenolic compounds in any plant species shows good antioxidant activity [38]. The results of the CUPRAC assay reveals that the LFD and plant extracts show high level of antioxidant potential. The antioxidant activity is concentration dependent, i.e., with an increase in concentration of LFD there is also an increase in the reduction of copper ions in the CUPRAC assay. Another approach for anti-Alzheimer’s is by inhibiting the acetylcholinesterase enzyme because it breaks down acetylcholine (neurotransmitter) into acetic acid and choline. Acetylcholine is necessary for the normal functioning of brain [39]. The results of the present study reveal that the LFD shows 60.30% and 24.04% inhibition against AChE and BChE, respectively.

Cancer is a leading cause of death worldwide. The available anticancer drugs have poor selectivity and severe side effects [40]. In the present study, the cytotoxic potential of methanol, dichloromethane extract of *C. lancifolius,* and novel compound LFD against normal and cancerous cells is determined. The results of this study reveal that LFD significantly increased the death of murine lymphocytic leukemia and human breast cancer cells when compared to ASK and Hek293 as normal cells. CLD and CLM showed good cytotoxic activity against all the cancerous cell lines. Cytotoxic ED_50_ concentration is considered active if ED_50_ is less than 4 µg/mL and 20 µg/mL for extracts or pure compounds, respectively.

HSV-1 causes a range of infections in the mouth, mucous membranes, eyes, and pharynx, and is more common in immunocompromised patients [7]. In the present study, the virucidal effect of LFD and plant extracts (CLD, CLM) against HSV-1 was determined. The LFD shows high % inhibition at 26%. The antibacterial and antifungal activities of the extracts from the test samples in terms of minimum inhibitory concentrations (MIC) and diameters of inhibition zones are reported in Table 8. In the present study, the novel compound LFD had high activity against *S. mutans, C. albicans,* and *C. neoformans,* and moderate activity against *B. subtilis, B. laterosporus,* and *S. typhi.*

## 5. Conclusions

This study was designed to evaluate the anti-Alzheimer’s, anti-cancer, and cytotoxic potential of novel lancifolamide (LFD) isolated from *C. lancifolius* methanol extract. The anticholinesterase and antioxidant activities of the extracts along with isolated lancifolamide were examined. The possible mechanism of action for anti-Alzheimer’s, anti-viral, and cytotoxic effects of LFD is determined by our docking study. Results of the experimental studies exhibited isolated compounds which showed anti-Alzheimer’s, antiviral, and cytotoxic effects.

## Figures and Tables

**Table 1:** ^13^C-NMR (125 MHz) or Chemical shift (^1^H-NMR 500MHz) spectral data of “LFD”.

Carbon No.	Multiplicity DEPT	C^13^–NMR (δ)	Proton NMR	J. Value
C–1	C	109.50	-	-
C–2	C	138.20	-	-
C–3	CH	138.54	6.636 d	(J = 3.2, Hz, H-1)
C–4	CH	109.24	6.633 d	(J = 2.6, Hz, H-2)
C–5	C	108.20	-	-
C–6	CH	109.40	-	-
C–7	C	109.91	-	
C–8	C	113.55	-	-
C–9	CH	139.35	-	-
C–10	C	139.92		
C–11	C	148.72	-	-
C–12	C	105.55	6.857 d	(J = 3.1, 2.4 Hz, H-2)
C–13	C	110.22	-	-
C–14	C	152.90	-	-
C–15	OCH_3_	54.45	3.347 s	(J = 4.2 Hz)
C–16	OCH_3_	59.20	3.984 s	(J = 5.5 Hz)
C–17	OCH_3_	60.55	4.580 s	(J = 4.4 Hz)
C–18	OCH_3_	60.55	4.635 s	(J = 4.4 Hz)

**Table 2:** Docked ligands Surflex scores LFD, GLM and ACV in distinct protein binding site.

Protein	Ligand	CScore *^a^*	Crash Score *^b^*	Polar Score *^c^*	D Score *^d^*	PMF Score *^e^*	G Score *^f^*	Chem Score *^g^*	Amino Acid Interaction
**Acetylcholine**	**LFD**	6.63	−1.81	1.17	−148.754	−13.63	−217.817	−17.05	G120, G121, G122, E202, H447,
	**GLM**	5.64	−1.70	0.00	−132.573	−5.102	−256.580	−23.36	G120, S125, Y133,
**DNA polymerase**	**ACV**	10.32	−0.55	6.59	−105.060	−33.78	−175.87	−0.087	Y101, E225, R176, M128,
	**LFD**	9.09	−3.83	4.17	−159.13	−13.86	−308	−22.11	R176, R222, M128, Y132
**BCL-xl**	**LFD**	6.72	−1.18	0.10	−129.044	−51.80	−255.476	−20.96	S106, E129, R132, R139,
**BCL-2**	**LFD**	4.18	−0.56	1.89	−86.197	−0.466	−134.869	−14.15	Q118, A149
**Caspase-3**	**LFD**	4.78	−1.05	2.01	−92.743	29.101	−148.402	−7.972	H121, G122, E123, T166,
**NF-kb**	**LFD**	3.86	−4.33	2.73	−169.628	114.664	−300.937	−22.57	Y319, D321, S329
**TNF-α**	**LFD**	2.77	−1.13	1.57	−79.118	−63.603	−129.618	−17.08	Y59, Q149

***^a^* CScore**; consensus scoring utilizes multiple scoring function types to rank ligands affinity, ***^b^* Crash**-score divulging inappropriate penetrating binding site, ***^c^* Ligand polar** region, ***^d^* D-score** exhibiting complex (ligand–protein), hydrogen bonding, interior energies i.e., ligand to ligand, ***^e^* PMF-score** specifying Helmholtz free interaction energies for pairs protein to ligand atom (Mean Force Potential or PMF), ***^f^* G-score** for interactions such as Van der Waals or charge between ligand or protein, ***^g^* Chem-score** points for rotational entropy, lipophilic contact, hydrogen bonding with intercept term.

**Table 3:** IC_50_ values of the *C. lancifolius* extracts (μg/mL). Mean ± SD (n = 3).

Antioxidant Assays	CLD *	CLM **	LFD ***	BHT	BHA	α-Tocopherol
DPPH	NA	162.21 ± 4.56	NA	74.59 ± 1.98	16.19 ± 0.56	33.09 ± 0.68
ABTS	NA	69.16 ± 2.29	173.15 ± 6.73	18.35 ± 0.67	12.66 ± 0.47	19.35 ± 0.21
β-Caroten	139.12 ± 0.20	NA	140.91 ± 4.85	6.24 ± 0.39	5.06 ± 0.37	0.53 ± 0.73

* CLD: Dichloromethane extract of *C. lancifolius.* ** CLM: Methanol extract of *C. lancifolius.* *** LFD: Lancifolamide.

**Table 4:** CUPRAC assay results. Mean ± SD (n = 3).

Concentrations	10 μg/mL	25 μg/mL	50 μg/mL	100 μg/mL
CLD *	0.13 ± 1.20	0.17 ± 0.42	0.22 ± 1.64	0.30 ± 2.35
CLM **	0.13 ± 2.62	0.21 ± 1. 05	0.32 ± 0.65	0.49 ± 0.30
Lancifolamide	0.11 ± 2.54	0.19 ± 2.10	0.26 ± 1.85	0.41± 2.11
BHA	1.22 ± 0.35	1.60 ± 0.62	2.10 ± 0.55	2.39 ± 0.15
BHT	1.49 ± 1.15	1.78 ± 1.36	2.04 ± 0.70	2.42 ± 0.45
α-Tocopherol	0.29 ± 1.20	0.52 ± 0.90	0.68 ± 0.25	1.28 ± 1.10

* CLD: Dichloromethane extract of *C. lancifolius.* ** CLM: Methanol extract of *C. lancifolius.*

**Table 5:** Cytotoxicity of pure compounds or crude extracts from *C. lancifolius.* Mean ± SD (n = 3).

Crude Extracts/Pure Compounds	Cancer Cells				Normal Cells	
	P-388	Col-2	MCF-7	Lu-1	ASK	Hek293
Dichloromethane	8.15 ± 2.11	7.24 ± 2.50	1.19 ± 1.40	11.65 ± 1.95	9.04 ± 1.65	5.32 ± 0.54
Methanol	2.06 ± 2.01	8.14 ± 3.10	0.85 ± 2.66	7.64 ± 0.45	5.40 ± 0.30	<4.00
Compound (lancifolamide)	2.01 ± 3.15	NR *	0.72 ± 3.92	NR *	2.40 ± 2.55	1.55 ± 1.02
Ellipticine (Positive control)	0.42 ± 0.03	0.53 ± 0.11	0.36 ± 0.92	0.22 ± 0.73	0.23 ± 0.70	0.57 ± 1.63

* NR: No response.

**Table 6:** Assay of Anticholinesterase at 200µg/mL Mean ± SD (n = 3).

Sample	Inhibition % against BChE	Inhibition % against AChE	IC_50_ Values in μg/mL of AChE
CLD *	09.04 ± 0.11	69.10 ± 3.32	25.14 ± 2.62
CLM **	5.73 ± 0.07	62.45 ± 2.53	29.55 ± 2.10
Lancifolamide	24.04 ± 1.93	60.30 ± 2.86	34.62 ± 1.40
Galantamine	76.25 ± 4.51	75.20 ± 2.78	13.20 ± 0.15

* CLD: Dichloromethane extract of *C. lancifolius.* ** CLM: Methanol extract of *C. lancifolius.*

**Table 7:** Anti-HSV-1 extract activities along with lancifolamide compound secluded from *C. lancifolius*. Mean ± SD (n = 3).

Compound	IC_50_ (mg/mL)	IP (%)
CLM	>100	17 ± 1.08
CLD	>100	13 ± 0.86
Lancifolamide	>100	26 ± 1.89

**Table 8:** Antimicrobial and antifungal potential of extracts and lancifolamide.

**Bacteria**	**Microorganisms**	**Activity**	**CLD**	**CLM**	**Lancifolamide**	**RA**
	*B. subtilis*	MIC	0.275	0.275	0.275	7.8
		MBC	0.55	0.55	0.55	15.6
	*S. mutans*	MIC	1.10	2.20	1.10	7.8
		MBC	2.20	4.40	2.20	15.6
	*B. laterosporus*	MIC	0.137	0.275	0.137	3.9
		MBC	0.275	0.55	0.275	7.8
	*S. typhi*	MIC	0.275	0.55	0.137	3.9
		MBC	0.55	1.10	0.275	7.8
**Fungi**	*C. albicans*	MIC	0.55	2.20	0.55	NT
		MFC	1.10	4.40	1.10	NT
	*C. neoformans*	MIC	0.275	0.275	0.275	NT
		MFC	0.55	0.55	0.55	NT

## Data Availability

Not applicable.
